# Design and validation of a supragenome array for determination of the genomic content of *Haemophilus influenzae* isolates

**DOI:** 10.1186/1471-2164-14-484

**Published:** 2013-07-17

**Authors:** Rory A Eutsey, N Luisa Hiller, Joshua P Earl, Benjamin A Janto, Margaret E Dahlgren, Azad Ahmed, Evan Powell, Matthew P Schultz, Janet R Gilsdorf, Lixin Zhang, Arnold Smith, Timothy F Murphy, Sanjay Sethi, Kai Shen, J Christopher Post, Fen Z Hu, Garth D Ehrlich

**Affiliations:** 1 Center for Genomic Sciences, Allegheny Singer Research Institute, Allegheny General Hospital, 320 East North Avenue, 11th Floor, South Tower, Pittsburgh, PA 15212, USA; 2 Department of Biological Sciences, Carnegie Mellon University, Pittsburgh, PA, USA; 3 Department of Microbiology and Immunology, Drexel University College of Medicine, Allegheny Campus, Pittsburgh, PA, USA; 4 Department of Epidemiology, University of Michigan School of Public Health, Ann Arbor, MC, USA; 5 Department of Pediatrics and Communicable Diseases, University of Michigan School of Public Health, Ann Arbor, MC, USA; 6 Center for Childhood Infections, Seattle Children’s Hospital Research Institute, Seattle, WA, USA; 7 Department of Medicine, University at Buffalo, State University of New York, Buffalo, NY, USA; 8 Department of Otolaryngology Head and Neck Surgery, Drexel University College of Medicine, Allegheny Campus, Pittsburgh, PA, USA

## Abstract

**Background:**

*Haemophilus influenzae* colonizes the human nasopharynx as a commensal, and is etiologically associated with numerous opportunistic infections of the airway; it is also less commonly associated with invasive disease. Clinical isolates of *H. influenzae* display extensive genomic diversity and plasticity. The development of strategies to successfully prevent, diagnose and treat *H. influenzae* infections depends on tools to ascertain the gene content of individual isolates.

**Results:**

We describe and validate a *Haemophilus influenzae* supragenome hybridization (SGH) array that can be used to characterize the full genic complement of any strain within the species, as well as strains from several highly related species. The array contains 31,307 probes that collectively cover essentially all alleles of the 2890 gene clusters identified from the whole genome sequencing of 24 clinical *H. influenzae* strains. The finite supragenome model predicts that these data include greater than 85% of all non-rare genes (where rare genes are defined as those present in less than 10% of sequenced strains). The veracity of the array was tested by comparing the whole genome sequences of eight strains with their hybridization data obtained using the supragenome array. The array predictions were correct and reproducible for ~ 98% of the gene content of all of the sequenced strains. This technology was then applied to an investigation of the gene content of 193 geographically and clinically diverse *H. influenzae* clinical strains. These strains came from multiple locations from five different continents and Papua New Guinea and include isolates from: the middle ears of persons with otitis media and otorrhea; lung aspirates and sputum samples from pneumonia and COPD patients, blood specimens from patients with sepsis; cerebrospinal fluid from patients with meningitis, as well as from pharyngeal specimens from healthy persons.

**Conclusions:**

These analyses provided the most comprehensive and detailed genomic/phylogenetic look at this species to date, and identified a subset of highly divergent strains that form a separate lineage within the species. This array provides a cost-effective and high-throughput tool to determine the gene content of any *H. influenzae* isolate or lineage. Furthermore, the method for probe selection can be applied to any species, given a group of available whole genome sequences.

## Background

The sequencing of multiple strains from single bacterial species has revealed extensive genomic diversity within species [1-10]. This variability is observed as single nucleotide polymorphisms (allelic differences) as well as extensive differences in gene possession [11,12]. Studies of strain variability within species have led to the definition of the supragenome or pangenome as the full complement of genes encountered within a species [1,11,13]. The supragenome is composed of the core genome, i.e. the set of genes present in all the strains of the species, and the distributed genome (also known as dispensable or accessory genomes) i.e. genes present in only a subset of strains. In a few species, notably *Mycobacterium tuberculosis*, all strains have a highly conserved gene content such that ~90% of genes are present in the core genome [7]. However, for the vast majority of bacterial species so examined the distributed genome is larger than the core. For example the genomic complements of the strains within the species *Bacillus subtilis*, *Escherichia coli*, and *Gardnerella vaginalis* are highly variable with the core genome making up only one third or less of the supragenome [7,14,15]. Differences in gene content across isolates account for differences in microbial functional activities, such as biofilm formation, pathogenic potential or antimicrobial resistance [12,16-19].

In addition to extensive diversity, comparative whole genome sequencing of multiple strains from the same species has revealed evidence of widespread horizontal gene transfer (HGT) among strains and even related species [4,20,21]. Gene exchange is a common and evolutionarily safe strategy, as opposed to point mutations, for bacteria to acquire novel gene combinations for adaption to environmental stresses, novel conditions, or new niches [12]. Thus, the species' supragenome represents the complete genetic repertoire from which individual isolates develop genomic variability as they exchange DNA. Knowledge of the naturally-occurring gene combinations is critically important for developing strategies for prevention, diagnosis, and treatment of bacterial infections considering that species-level information, such as are currently reported clinically, cannot distinguish between commensal and highly pathogenic strains of the same species.

Hybridization gene arrays provide a cost-effective and high-throughput means to investigate gene content. Most existing arrays, however, are based on the gene content of a single strain or a small number of reference strains with the addition of additional alleles for a few known highly variable loci, and thus, are not ideal to identify overall gene content from isolates of a diverse species. To overcome these limitations, we have developed a supragenome array capable of identifying over 85% of the non-rare (V > 0.1) genes (those most likely to be clinically important). While this analysis is focused on a single species, the design strategy can be applied to any bacterial supragenome.

*H. influenzae* is a gram-negative bacterium that colonizes the human nasopharynx, as a commensal organism, but acts as an opportunistic pathogen upon gaining access/entry to other body sites. Routine immunization against the highly virulent serotype b form of Hi (Hib), initiated in the 1980’s, has been very effective in reducing the incidence of *H. influenzae* sepsis, meningitis, and epiglotitis in the developed world [22]. In the post-Hib vaccine era, non-typeable *H. influenzae* (NTHi) continue to cause infections of the respiratory tree including otitis media (OM), conjunctivitis, sinusitis, pneumonia, and bronchitis especially in patients with chronic obstructive pulmonary disease (COPD); as well as playing a role in early colonization of the lower respiratory tracts of children with cystic fibrosis [23,24]. Over the last decade, Tsang and colleagues have documented an increase in the number of invasive NTHi [25-27]. Improved understanding of the bacterial factors that contribute to infection in various human niches is needed to design new strategies for their treatment or prevention.

## Methods

### Whole genome sequencing and assembly

A total of 24 *H. influenzae* whole genome sequences (WGS) were prepared or obtained for this study (Table 1). These included: 1) the nine NTHi strains previously sequenced using a 454 Life Sciences GS20 sequencer at the Center for Genomic Sciences (CGS) and used to develop the Finite Supragenome Model [2]; 2) ten additional CGS-sequenced NTHi strains prepared using one or more of the 454 Lifescience's technology platforms, including the GS20, FLX and Titanium as described [2,5,7] (Table 2); 3) the 4 NTHi genomes sequenced by others [2,28-30]; and 4) an Hib WGS (http://www.ncbi.nlm.nih.gov/genome/165?project_id=86647)[31]. All CGS-derived genomes were assembled using Newbler, as described [2].

**Table 1:** ***H. influenzae *strains used in design of CGH array**

**Strain name**	**# of Clusters**	**# of Coding sequences**	**Genome size (mb)**	**GC Content(%)**	**Clinical origin**	**Geographic origin**	**GenBank BioProject numbers**	**Reference**
22.1-21	1967	2220	1.89	38.03	Nasopharynx	Ann Arbor, MI	PRJNA54389	[32]
22.4-21	1957	1975	1.85	38.04	Nasopharynx	Ann Arbor, MI	PRJNA16396	[32]
22.1-24	1973	1915	2.1	37.91	Nasopharynx	Ann Arbor, MI	PRJNA29373	[32]
3655	1991	1951	1.88	38.02	Blood	San Diego, CA	PRJNA54385	[33]
6P18H1	2015	1952	1.91	38.1	COPD	Buffalo, NY	PRJNA55127	[34,35]
7P49H1	1897	1801	1.83	37.85	COPD	Buffalo, NY	PRJNA55129	[34,35]
PITT AA	2003	1973	1.88	38.12	COME, tube replacement	Children's Hospital, Pittsburgh, PA	PRJNA54391	[36]
PITT BB	1922	1892	1.83	37.99	COME, tube replacement	Children's Hospital, Pittsburgh, PA	PRJNA16402	CGS
PITT CC	1915	1892	1.82	37.98	COME, tube replacement	Children's Hospital, Pittsburgh, PA	PRJNA18099	CGS
PITT DD	1865	2900	1.78	37.89	COME, tube replacement	Children's Hospital, Pittsburgh, PA	PRJNA16392	CGS
PITT EE	1848	1870	1.81	38.04	COME, tube replacement	Children's Hospital, Pittsburgh, PA	PRJNA58591	[36]
PITT GG	1966	2097	1.89	38.01	AOM, ottorhea	Children's Hospital, Pittsburgh, PA	PRJNA58593	[36]
PITT HH	1948	1968	1.84	38.00	COME, tube replacement	Children's Hospital, Pittsburgh, PA	PRJNA54393	[36]
PITT II	2057	2038	1.95	38.01	AOM, ottorhea	Children's Hospital, Pittsburgh, PA	PRJNA54395	[36]
PITT JJ	2054	2293	1.97	38.03	COME, tube replacement	Children's Hospital, Pittsburgh, PA	PRJNA18103	CGS
NML20	1838	1757	1.78	37.91	Blood	Manitoba, Canada	PRJNA29375	CGS
R1838	1900	1813	1.82	38.01	Blood	Papua New Guinea	PRJNA29377	CGS
R3021	2017	1977	1.88	37.96	Nasopharynx	Seattle, WA	PRJNA54397	[29]
R393	2109	2175	2	38.15	Sputum isolate	Malaysia	PRJNA29379	CGS
86-028NP	2001	1925	1.91	38.15	Nasopharynx	Nationwide Childrens Hospital, Columbus, OH	PRJNA58093	[30]
B10810	2044	2007	1.98	38.1	Meningitis	United Kingdom	PRJNA86647	Sanger
R2846	1856	1803	1.82	38.02	OME	Seattle, WA	PRJNA191921	[29]
R2866	2017	1929	1.93	38.08	Blood	Seattle, WA	PRJNA161923	[29]
RD KW20	1892	1874	1.83	38.15	Laboratory Strain	Columbia University	PRJNA57771	[28]
**Avg**	1960.5	1999.875	1.8825	38.02	n/a	n/a	n/a	n/a

**Table 2:** Whole genome sequencing summary

**Strain name**	**# 454 Contig**	**# Contigs after closure**	**Coverage**	**# Assembled reads**	**Avg read length**	**Platform**
**PITT BB**	110	6	18.6	321633	106	GS20
**PITT CC**	43	13	20.5	347798	107.5	GS20
**PITT DD**	199	33	29.7	527939	100	GS20
**PITT JJ**	71	11	23.5	434215	106.7	GS20
**NML20**	41	18	24.9	409200	108.6	GS20
**R1838**	42	18	26.7	446399	109	GS20
**R393**	156	47	18.1	333127	108.6	GS20
**22.1-24**	270	17	17.6	342141	107.8	GS20
**6P18H1**	50	28	30.9	233434	250	FLX
**7P49H1**	36	19	28.7	208570	250	FLX

### Identification of coding sequences for the 24 WGS strains

The 24 genomes were submitted in parallel to the Rapid Annotations with Subsystems Technology (RAST) annotation service [37].

### Gene clustering algorithm

A complete description of the algorithms used to create the gene clusters and subclusters is given by Hogg et al. [2]. Briefly, tfasty36 (Fasta package, version 3.6) was used for six-frame translation homology searches of all predicted proteins against all possible translations [38]. These results were parsed to select for all coding sequences that were above a threshold based on a selected identity and length. For grouping into clusters the threshold was set to 70% identity over 70% of the length of the shorter sequence. This single linkage algorithm will thus link together genes that are split in some strains but fused in others, so that it works well for dealing with gapped genome data. For grouping into subclusters, each gene in a cluster was compared to all other genes in the same cluster, and sequences with at least 95% identity over 95% of the length of the shorter sequence were grouped together.

### Design of the SGH array

The SGH Array was designed using the WGS's of the 24 *H. influenzae* strains (that represented the set of all *H. influenzae* genomes available at the time of construction). To design probes that recognize all of the known *H. influenzae* genes, the 47,997 coding sequences from these genomes were divided into 3100 clusters (Table 3). Each cluster contains sequences that are at least 70% identical over 70% of the length; this strategy groups together the orthologues across strains, as well as highly related genes within strains. Of these clusters, 1538 contained 38,184 sequences shared by all strains (core clusters); while 1562 contained 9,813 sequences present in a subset of 1 to 23 strains (distributed clusters). Many of these clusters contain multiple allelic variants, such that if probes were designed to only one representative sequence from each cluster they may not hybridize to all the alleles. To ensure the selection of probes that will hybridize to all known alleles, each cluster was further split into subclusters that grouped all sequences together that are 95% identical over 95% of the length of the shorter sequence. There were 4536 subclusters, of which 2350 corresponded to core sequences and 2186 corresponded to distributed sequences.

**Table 3:** Gene clustering and probe design

	**# Sequences**	**# Clusters**	**# Clusters with probes**	**# Subclusters**	**# Subclusters with probes**	**# Individual probes**	**# Probes (account duplication)**
Distributed Set	9813	1562	1465	2186	2008	25267	50534
Core Set	38184	1538	1425	2350	2044	6040	12080
**All Clusters**	**47997**	**3100**	**2890**	**4536**	**4052**	**31307**	**62614**
Negative Controls	185	n/a	n/a	n/a	n/a	185	370
Nimblegen Controls	n/a	n/a	n/a	n/a	n/a	9053	9053
**Total**	**48182**	**3100**	**2890**	**4536**	**4052**	**40545**	**72037**

**Figure 1:**
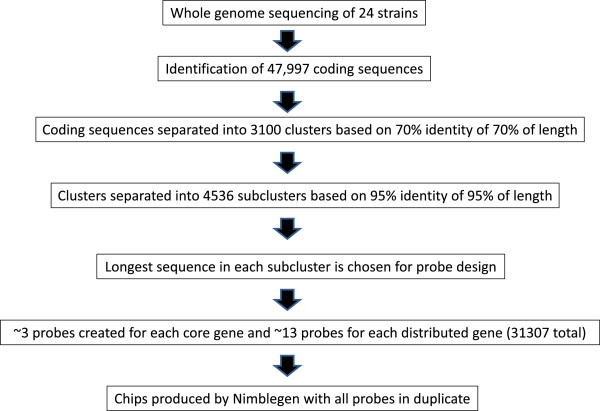
Schematic illustrating the stepwise strategy used to design the SGH array.

Once the coding sequences were organized into subclusters of highly related sequences, we used the longest sequence from each subcluster to create probes 60 bases in length. We designed 25267 different probes to 2008 of 2186 distributed subclusters (average of 12.5/subcluster), and 6040 probes to 2044 of the 2350 core subclusters (average of 2.95/subcluster) (Table 3). This set covers 2890 of the 3100 clusters. A portion of the subclusters, 178 of the distributed and 306 of the core, were not amenable to probe design in most cases due to reasons such as short sequence, homopolymer runs, or only low complexity sequence. We also added 185 negative control probes, designed from *S. pneumoniae* sequences. All probes were placed on the final array in duplicate (Table 3, Additional file 1: Table S1, Figure 1).

### Hybridization array probe design

*H. influenzae* specific: The longest sequence from each subcluster was used as a template to create probes of 60 bases in length. A set of 20 potential probes per subcluster was created by Nimblegen (Roche; Madison WI) using their software. The goal was to design ~13 probes corresponding to each distributed subcluster and ~3 probes corresponding to each core subcluster. Probes were ranked based on their specificity to clusters, specificity to subclusters, and probe-design parameters. To determine cluster and subcluster specificity each probe was compared using BLASTN to a database of all *H. influenzae* coding sequences from the 24 WGS's. The ideal probes have high scoring hits to all members of their subcluster, and no hits outside the cluster. Hits were ranked such that probes with the best rank contained high scoring hits to all members of the subcluster and lower scores to members of other subclusters. Next ranked were the probes with hits to members of the same subcluster as well as other subclusters. The worst score was to probes that only recognized a subset of the sequences in the same subcluster. Probes with similar subcluster specificity, were further ranked using the Nimblegen ranking algorithm, which accounts for uniqueness, distribution within the sequence (aimed at an even distribution), and probe manufacturing parameters. The negative controls were selected by using BLASTN to query *S. pneumoniae* genes from 44 strains [4] against a database of all the coding sequences from the 24 *H. influenzae* genomes. The goal was to choose *S. pneumoniae* genes that have no homologues in *H. influenzae*, thus we selected a set of relatively long genes (> 500 bp) with only very low scoring hits (e-value above 1e-4). 185 sequences were selected, and one probe was designed to each one of these. Nimblegen generates a set of 9053 random control probes that serve as negative background hybridization controls. Alignment and tracking probes that bind to oligos added during hybridization allow the image analysis software to correctly determine probe grid positions as well as detect mixing between samples. These oligos were also used to determine the hybridization evenness over the entire probe covered area.

### DNA extraction for hybridization

Overnight NTHi cultures were grown in 30 mL supplemented BHI broth and the bacteria were pelleted at 4000 rpm for 5 minutes. Genomic DNA (gDNA) was extracted from the pellet using the standard 24:1 Chloroform/Isoamyl alcohol method and stored in 1X TE buffer [39]. Quality control was performed using the Nanodrop 1000, as well as running ~1 μg on a 1% TAE gel to observe molecular weight. If necessary, gDNA was treated a second time with RNaseA and Proteinase K, then reprecipitated to ensure sample purity [40].

### DNA labeling for hybridization

gDNA samples were labeled using the Nimblegen One Color DNA Labeling Kit (NimbleGen Arrays User’s Guide: Gene Expression Arrays Version 6.0). Briefly, DNA samples were heated to 98°C for 10 minutes in the presence of Cy3 labeled random nonomers and then cooled rapidly. This reaction was then incubated at 37°C for 2 hours with dNTPs and Klenow fragment to complete labeling. Finally, the labeled DNAs were subjected to an isopropanol precipitation to get rid of unincorporated nucleotides and primers.

### Hybridization and washing

Labeled DNA was prepared for hybridization by lypophilizing 2 μg in a SpeedVac and resuspending in sample tracking solution (a different tracking solution is used for each sample). The sample was then mixed with the components of the Nimblegen Hybridization Kit (Hybridization buffer, component A, and alignment oligo) and incubated at 95°C for 5 minutes before being loaded onto the NimbleGen microarray. The loading was carried out by pipetting the sample into a custom-built mixer that is adhered to the surface of the array. This assembly was then loaded into the Nimblegen Hybridization station and incubated for 18 hours. After incubation, arrays were washed using the NimbleGen Wash Buffer Kit and dried using the NimbleGen Slide Dryer.

### Array scanning

Arrays were scanned using a Molecular Devices Axon GenePix 4200AL. Images were processed using Nimblegen NimbleScan software.

### Testing accuracy of 24 input strains

The presence/absence profile for each of the 2890 gene clusters from the *H. influenzae* supragenome that were represented on the array was compared to the gene possession data from each of the 24 WGS’d strains as an objective means to determine the accuracy of the arrays. Presence/absence for the array was determined as described below in data analysis.

### Testing accuracy of CZ4126/02

To establish whether the clusters identified by the array matched the whole genome sequencing data we used BLASTN. For each cluster a representative sequence from one of the original 24 genomes was selected. This representative was compared to the whole genome sequence of a 25th sequenced NTHi strain, CZ4126/02, GenBank accession number PRJNA189674 (Janto unpublished). If a hit was identified above the e-value threshold of 1e-20, the cluster was considered present in the genome. If no hits were observed at this threshold, the cluster was considered absent.

### Data analyses

Data were processed and normalized within arrays using a Robust Multichip Average (RMA) algorithm and quantile normalization using the NimbleScan software. Raw data were converted into cluster presence or absence by applying an expression threshold (set to 1.5X the median background value using a log_2_ scale). To determine intraslide consistency a Student T distribution analysis was used. A cluster was considered present if the signal for any of its subclusters was above the threshold and the p-value for the probe set was below 0.05. Note that the subclustering data cannot be used to confidently determine which allele is present, since small numbers of variations between a probe and sequence may still allow hybridization (the extent depends on the actual sequence).

### Tree building

The ‘ape’ package in the ‘R’ environment was used to build a distance matrix based on the presence of clusters (as determined by SGH Array, or WGS when array data was not available) using the binary setting [41]. A tree was generated from the distance matrix using the nearest neighbor method and visualized with FigTree v1.3.1 (available at http://tree.bio.ed.ac.uk/software/figtree/)[42].

### Cost and time analysis

The costs incurred per sample are approximately $110. Samples can be processed in four days from culture to output.

## Results

### Genome sequencing and annotation

Twenty-four *H. influenzae* WGS's were utilized in the construction of a species-level supragenome hybridization (SGH) array (Table 1). At the start of this study 14 *H. influenzae* WGS's were available; the 13 described in Hogg et al. [2] , which were a lab strain (Rd), four nasopharyngeal isolates (86-028NP, R3021, 22.4-21, and 22.1-21), a blood isolate (R2866), and seven strains isolated from the middle ears of pediatric patients. Specifically, one acute otitis media isolate (3655), four chronic otitis media isolates (R2846, Pitt AA, Pitt EE, Pitt HH), and two ottorheic isolates (Pitt GG and Pitt II). There was also a type b strain (10810) sequence available through the NCBI microbial genome database [31]. To increase both the geographic and disease diversity of the sequenced strain set and to ensure that we had high coverage of all non-rare genes (V ≥ 0.1) at the species level as predicted by the Finite Supragenome Model [2,5], ten additional NTHi genomes were sequenced at the Center for Genomic Sciences (CGS) using 454 LifeSciences pyrosequencing (Table 2). These strains consisted of: four trans-tympanic isolates obtained from patients with chronic otitis media with effusion (COME) undergoing tube placement (PittBB, PittCC, PittDD, and PittJJ); two septic blood isolates (NML20 and R1838); three sputum isolates from patients with COPD (6P18H1, 7P49H1, and R393); and one additional NP isolate (22.1-24). Genome coverage levels ranged from 15.5 - 45.8 and the number of contigs obtained by the Newbler assembler from the pyrosequencing data was between 36 and 270 for the 19 CGS-sequenced strains. Gap filling using PCR and Sanger sequencing of the resultant amplicons was performed as described [3] to reduce the number of contigs/genome to between 1 (genome closure was achieved for two strains PITT EE and PITT GG) and 59 (PITT HH) for the 19 CGS sequenced strains. The average GC content for the ten newly sequenced strains was 37.98% and their average genome size was 1.85 Mb. These figures are nearly identical to the averages for the entire 24 strain set which averaged 38.02% GC, with an average genome length of 1.88 Mb. The final assemblies for the ten novel genomes have been deposited in GenBank, the accession numbers are: 22.1-24:PRJNA29373; 6P18H1: PRJNA55127; 7P49H1:PRJNA55129 ; PittBB:PRJNA16402; PittCC:PRJNA18099; PittDD:PRJNA16392; PittJJ:PRJNA18103; NML20:PRJNA29375; R1838:PRJNA29377, and R393:PRJNA29379.

### Identification of coding sequences for the 24 genomes

Using RAST [37] to annotate the 24 genomes we identified 47,997 coding sequences, with an average of 2000 per strain (Table 1). The annotations for the ten newly sequenced genomes were deposited in Genbank under the following accession numbers: 22.1-24:PRJNA29373; 6P18H1:PRJNA55127; 7P49H1: PRJNA55129;PittBB:PRJNA16402; PittCC:PRJNA18099; PittDD:PRJNA16392; PittJJ:PRJNA18103; NML20:RRJNA29375; R1838:PRJNA29377 and R393:PRJNA29379.

### Coverage of the supragenome

We applied the Finite Supragenome Model [2,5] to estimate the size of the supragenome based on the genomes from the 24 strains. Our model predicts a supragenome of 4547 clusters, 1485 core (32.67%) and 3062 distributed. However, 1806 (39.73%) of the distributed clusters are predicted to appear in less than 10% of strains and are considered rare genes, with the remaining 2741 clusters representing the distributed set present in at least 10% of strains. We extrapolate that the 2890 clusters represented on the array represent 63.5% of all *H. influenzae* clusters. Furthermore, the 2890 clusters include 2308 non-rare clusters, with the remaining 582 clusters being present in 2 or fewer of the 24 original strains. Thus ~ 85% (2308/2741) of the non-rare genes from the species supragenome are represented on this array.

### Accuracy of the array

**Figure 2:**
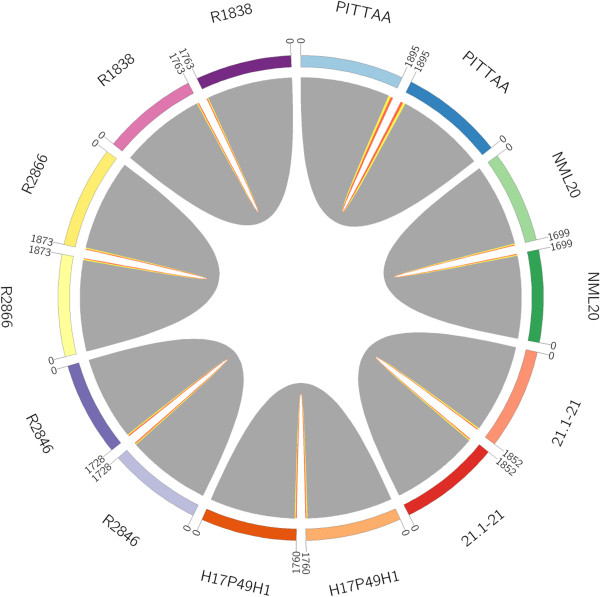
**Comparison between WGS data and SGH array data, as represented by a CIRCOS diagram.** Grey: gene clusters where both methods agree; yellow: negative on SGH array but positive for WGS; orange: positive on SGH array but negative for WGS. Paired numbers represent genes within each genome.

To investigate the accuracy of the SGH array the possession profile for all 2890 clusters on the array was compared between the array output and the whole genome sequence (WGS) data for 7 of the 24 genomes used for the array design. The genomes used for comparison were: PittAA, NML20, 22.2-21, Hi7P49HI, R2846, R2866, and R1838 (Table 4). For the clusters represented on the array, we calculated: 1) the false negatives (those that were not captured by the SGH array, but are present in the WGS, represented in yellow in Figure 2); and 2) the false positives (those that were captured on the SGH array but are absent in the WGS, represented in orange in Figure 2). The WGS was considered the gold standard, although this may not always be the case since not all of these genomes are closed and thus contain gaps. It is likely that at least a subset of false positives represent the sequences within these gaps. On average there were 25 (1.44%) false negatives and 19 (1.1%) false positives/genome. These results are summarized in Table 4 and visualized in the CIRCOS diagram in Figure 2, where the matches between both methods are in gray, and the false positive and negative predictions in orange and yellow, respectively.

**Table 4:** Comparison of SGH to Whole Genome Sequencing

**Strain**	**No of clusters based on supragenome analysis**	**Clusters represented on chip**	**Number in agreement between WGS and CGH**	**False negatives (CGH -, WGS +) [%]**	**False positives (CGH +, WGS -)[%]**
PittAA	2003	1849	1803	46 [2.49%]	46 [2.49%]
NML20	1838	1688	1665	23 [1.36%]	11 [0.65%]
22.1-21	1967	1811	1786	25 [1.38%]	14 [0.77%]
HI7P49HI	1897	1744	1724	20 [1.15%]	16 [0.92%]
R2846	1856	1708	1686	22 [1.29%]	20 [1.17%]
R2866	2017	1860	1837	23 [1.24%]	13 [0.70%]
R1838	1900	1748	1728	20 [1.14%]	15 [0.86%]
**CZ4126-02**		1702*	**1656**	**46 [2.7%]**	**39 [2.29%]**

* based on WGS of CZ4126-02, and not included in SGHChip design.

After construction of the array, the NTHi strain CZ4126/02 was also analyzed by both WGS and the SGH array (Table 4). Since its genomic sequence was not available when the array was designed, it served as an excellent test case to evaluate the accuracy of the array on new genomes. By analysis of the WGS it was determined that the array contained probes for 1702 of the CZ4126/02 clusters. Of these, 97% (2805/2890) of the clusters on the array were correctly predicted. Thirty nine clusters detected by the arrays were missing in the WGS; these could be actual false positives and/or genes present in the contig gaps. Consistent with some of these SGH-positive/WGS-negative genes being present in the WGS gaps, is the fact that many of the WGS-missing genes are found in contiguous groups in other WGS strains (e.g. 10 of these genes are present as an uninterrupted linkage group in Hi6P18H1). Forty six genes did not hybridize to the array yet had at least a portion of the gene present in the CZ4126/02 WGS as determined by a BLAST comparison. Interestingly, though, in most of these cases only a section of the sequence (not the full sequence) was present in the WGS suggesting this is an upper estimate of the number of false negatives. Finally, four genes that are unique to CZ4126/02 are missing in all 24 other WGS strains. Since these rare genes were not known at the time of SGH array design, there are no probes to identify their presence and they must be considered false negatives.

### Reproducibility of technical replicates within and across SGH arrays

**Figure 3:**
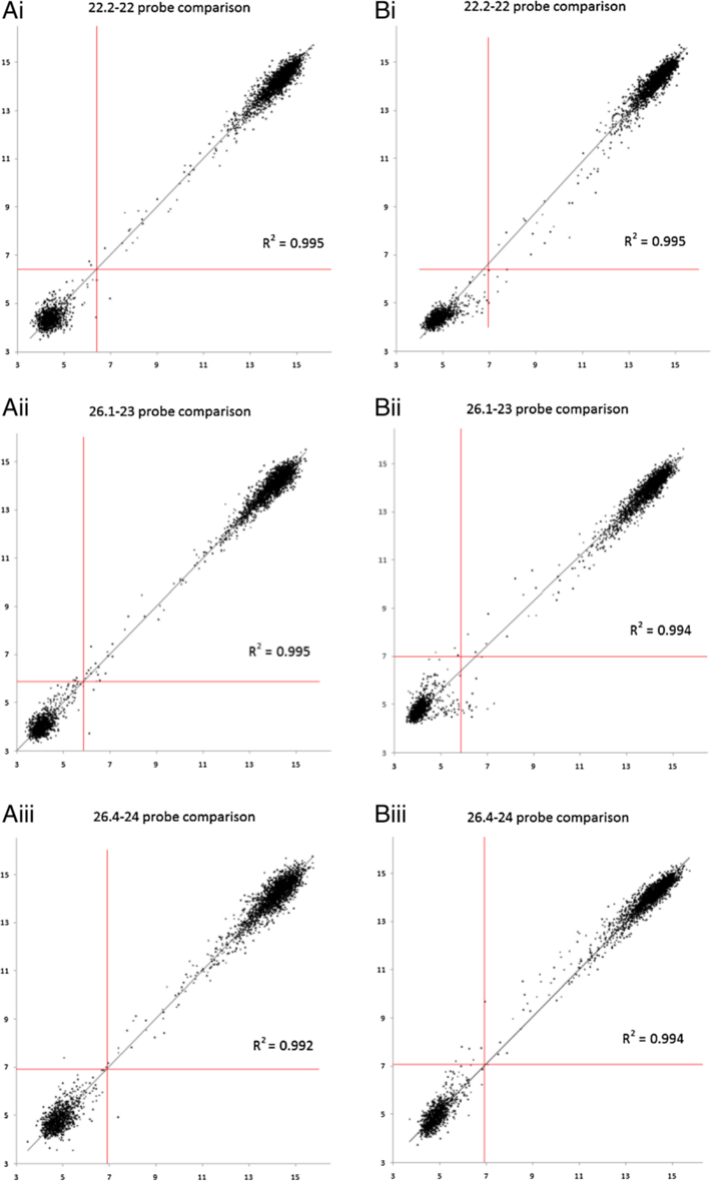
**Reproducibility of SGH array. (A)** X,Y Scatter plot of the hybridization values of duplicate probe sets for each cluster for a single sample within the same array. i: 22.2-22, ii: 26.1-23; iii: **(B)** X,Y scatter of the average hybridization values for each cluster of the same strains tested on separate arrays. i: 22.2-22, ii: 26.1-23; iii: 26.4-24. Red lines indicate thresholds used to define presence/absence of clusters. Hybridization values are displayed as log_2_. The subcluster with the highest value was chosen as the representative of the cluster.

The SGH Array was tested for reproducibility both between arrays and within the same array. Reproducibility within the same array was tested for strains 22.2-22, 26.1-23, and 26.4-24 by comparing hybridization results between the duplicate probe sets as each array has two copies of all *H. influenzae* and negative control probes (Figure 3Ai,ii,iii). Clusters appearing in the upper right quadrant are predicted to be present in both data sets of a comparison. As expected these represent the majority of the dots as an average genome has 1956 clusters and the array represents 2890 clusters in total. Clusters appearing in the lower left quadrant are missing in both data sets, and correspond to a subset of the distributed genes. For the upper left and lower right quadrant the hybridization value is above the threshold in one data set, and below in the other. The R^2^-values for the best-fit line of the X/Y scatter of each probe set for all three strains are > 0.99 suggesting very high fidelity of the probes within each array.

To investigate the reproducibility between SGH arrays, we used DNA isolated from the same three strains above (Figure 3Bi-iii). Each of these DNAs was subjected to separate labeling, hybridization, and analysis procedures for each of two SGH analyses. The number of clusters that yielded different possession profiles for the three strains 22.2-22, 26.1-23, and 26.4-24 respectively were 9, 18, and 6. Thus, the reproducibility of the data from the SGH arrays for these three strains was 99.69%, 99.38%, and 99.79% respectively. Note, that for a gene to be considered present, it must be above the threshold and the probe set must have a p-value < 0.05, thus there is not a perfect match between the thresholds illustrated in Figure 3 and the mismatched probes listed above.

### Analysis of the gene content of 210 *H. influenzae* strains

**Figure 4:**
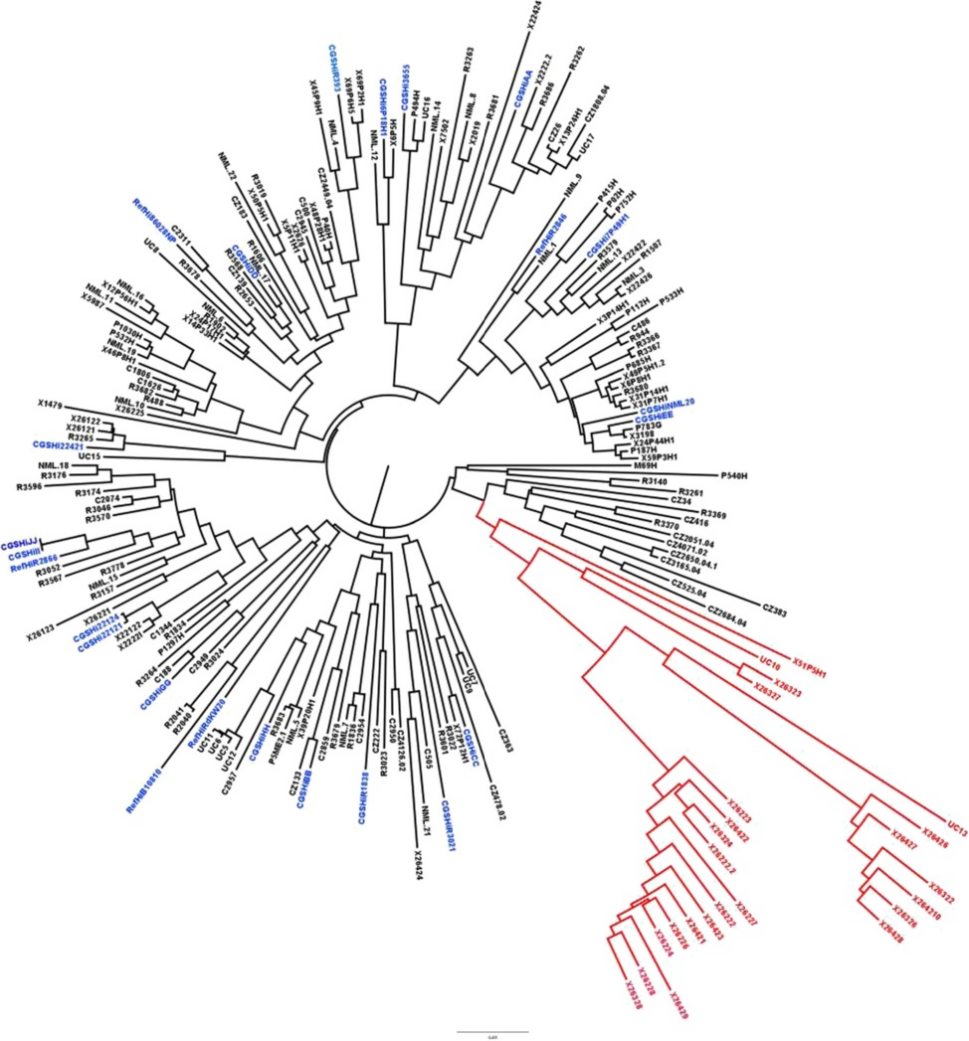
**Phylogenetic tree constructed using WGS data and SGH array data.** Blue: Strains with WGS used to design the array; Red: HDHi lineage.

**Figure 5:**
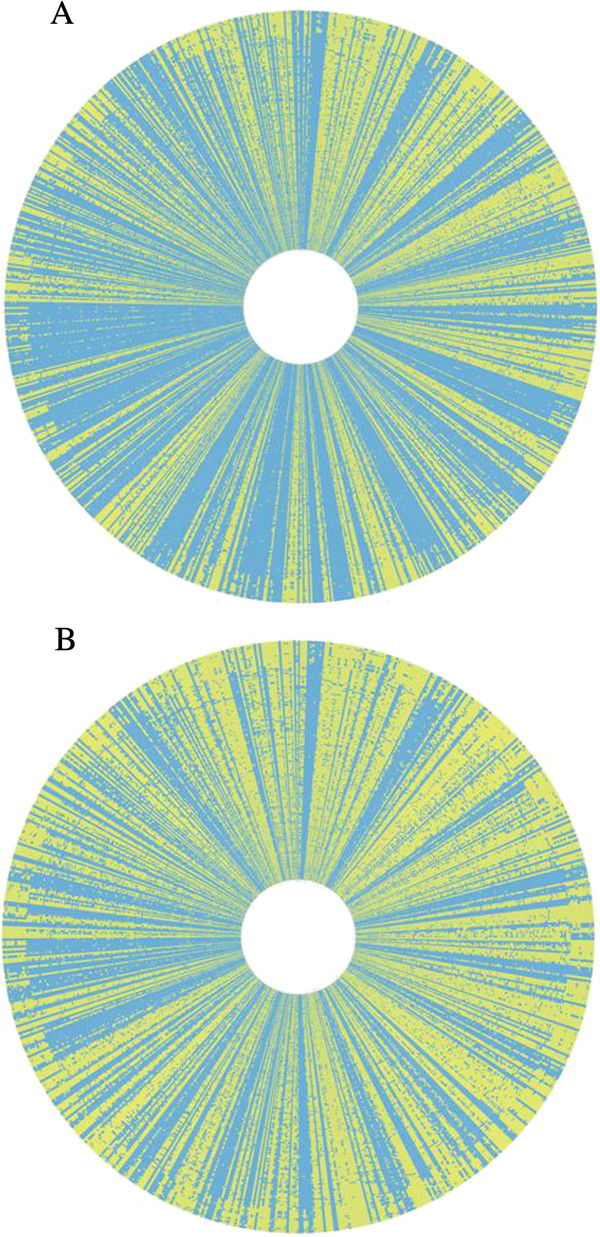
**CIRCOS representation of gene possession profiles.** Lines emanating from the center are gene clusters. Rings around inner circle represent one of the 210 strains analyzed; Blue represents presence of a cluster; Yellowrepresents absence of a cluster. **(A)** Analyses of all 2890 clusters on the chip. **(B)** Analyses of all 2212 distributed genes.

We next determined the gene content of 186 geographically and clinically diverse NTHi strains from collections around the world using the validated SGH arrays (Additional file 1: Table S1). These data were used to construct a distance-matrix tree which shows the relative relatedness of all strains based on essentially whole genome gene possession data (Figure 4). The tree shows the relative distances among all 210 (24 WGS + 186 SGH) genomically characterized *H. influenzae* strains. The 24 strains with WGS (colored blue in Figure 4) are distributed evenly around the tree indicating that they represent a broad sample of the species as intended. Surprisingly, of the 1538 clusters present in all 24 sequenced strains, only 678 (47%) were found to be present in all 210 strains. This number would suggest that only 23% of the genome is core, whereas we had previously predicted that the core would make up 47% of the supragenome [2]. The reason for this finding is that a previously unidentified lineage of 24 highly-related strains are all missing many of the core genes (red in Figure 4). If this distinct lineage is removed from the analysis then the core genome includes 1049 clusters. Even in this reduced set of 186 strains, 3 other strains (CZ383, P533H and R3262; all carriage strains isolated from the nasopharynx), each in a different lineage, are each missing over 50 core clusters based on the 24 WGS strains. Thus, it appears that it is not uncommon for strains and lineages to arise via substantial genomic deletions. At this point we don’t know if these strains have replaced these deleted genes with similar sized insertions or whether the genes are still present but have diverged in sequence such that they do not hybridize to probes on the SGH array. However, once these outliers are removed, 94.5% of the strains contain at least 98% of the 1425 clusters that were core in the 24 original strains. Thus the vast majority of these WGS core clusters are present in most of the strains. The extensive strain variability is visualized in Figure 5A and B; where each of the gene clusters is represented as a line radiating from the center, while each strain is represented as a concentric circle. Figure 5A illustrates the presence (blue) or absence (yellow) of each of the 2890 clusters for each of the 210 strains; where the 678 core genes are represented by radiating lines that are completely blue. Figure 5B represents only the 2212 distributed gene clusters. Thus, this figure illustrates how the distributed gene set ranges from very common (where the radiating line is mostly blue) to rare (where it appears as mostly yellow). Further, the 24 strains that are missing many core genes are displayed in the outermost circles, where their missing genes can be clearly visualized as lines that are blue in the innermost 186 circles and yellow in the 24 outer circles.

Each of the strains comprising the novel 24-strain divergent lineage, identified above, is missing between 82 and 274 genes previously considered core, suggesting that this lineage is either a subspecies or even a different species. We refer to this set of strains as the highly divergent *H. influenza*e (HDHi) (Table 5). Thus, the SGH array provides not only a high-throughput means to determine the gene content of any *H. influenzae* isolate, but also can serve as a tool to identify separate lineages and/or species. The classification of a strain as part of a separate species or lineage is based on the number of core genes represented in the new isolate. All *H. influenzae* strains share ~50% of the supergenome (the core gene set). If an isolate is missing a significant percentage of the core gene set, it strongly suggests it is part of a different species or a distantly related lineage.

**Table 5:** HDHi strains

**Strain**	**# NTHI core genes missing**	**Meet criteria for NTHi **[43]
26.3-22	274	No
UC13	274	NA
26.4-210	268	No
26.3-26	265	No
26.4-28	264	No
26.4-26	243	No
26.4-29	234	No
26.4-27	225	No
26.3-28	218	No
26.2-28	212	No
26.2-27	206	No
26.4-23	201	No
26.2-22	198	No
26.4-21	196	No
26.2-26	194	No
26.2-24	193	No
26.4-22	161	No
26.2-22.2	160	NA
26.2-23	148	No
26.3-24	148	No
51P5H1	105	NA
UC10	99	NA
26.3-23	97	Yes
26.3-27	82	Yes

### Correlation between gene content and virulence potential

The 186 NTHi strains used in this study were divided into virulent, commensal or unknown (Additional file 1: Table S1 and Additional file 2: Table S2). The virulent set consists of 117 strains isolated from sick patients, often from sites normally considered sterile, such as the blood, CSF, lung aspirates, and the middle ear. The commensal set consists of 65 strains isolated from the nasopharynx or throats of healthy individuals. Finally four strains were classified as unknown given lack of information or their lab origin (Rd KW20). The analysis was focused on NTHi, thus the 24 HDHi strains (23 commensal and 1 virulent) were not considered in this analysis.

A Fisher Exact test of the commensal versus virulent strains identified 149 genes, which are significantly enriched in one set versus the other (Additional file 2: Table S2). Specifically 28 genes are more likely to be found in the virulent strains, while 121 were found to be enriched in the commensal strains. The high number of genes associated with commensal strains is consistent with the existence of functional categories that promote non-pathogenic growth. The annotation of "hypothetical gene" is the most common for both sets of these differentially possessed genes, accounting for nearly half (73/149) of the gene calls and indicating that there is much new biology to be learned with regard to the type of symbiotic relationship that develops between host and bacteria. The most common characterized functional category in the entire set of genes are the methylases, consistent with an important role for DNA modification in the regulation of virulence. There are 6 and 4 unique methylase clusters in the sets of genes enriched among the commensal and virulent strains, respectively (Additional file 3: Table S3). Similarly, different clusters of TRAP-type C4-dicarboxylate transport system also are associated with either the virulent or pathogenic strain set (Additional file 2: Table S2).

Among the 28 genes enriched in the pathogenic strains are the virulence associated toxin-antitoxin gene systems which are widespread amongst prokaryotes. These systems each consist of two genes, one of which codes for a toxin which cleaves single stranded RNA (via a PIN domain), and the other which codes for the antitoxin (part of the antitoxin-MazE superfamily) [43]. Other annotations of the pathogenic-enriched gene set include a highly conserved locus related to glycerol-3-phosphate functional under anaerobic conditions. Finally, consistent with a role for surface proteins in host interactions; a putative surface protein is present in 25% of the virulent strains and only 12% of the commensals (it contains the Pfam DUF340 conserved domain which is of unknown function but contains a core of four predicted transmembrane regions).

The number of proteins associated with the commensal strains was much higher, corresponding to 121 genes. The gene cluster (representative member 6P18H1_1695) with the greatest p-value (1.3E-5) of all genes in either set is associated with 52% of commensals but only 20% of pathogenic strains (Additional file 2: Table S2). It is annotated as hypothetical, and does not contain any well characterized motifs. A search of the non-redundant database reveals homologues only in the Haemophilus genus (*H. influenzae* and *H. haemolyticus*). This set of genes also includes two putative transcriptional regulators, which are both short (under100 residues) and contain a helix turn helix domain (representative sequences are CGSHi22421_177 and CGSHi22421_1968). It will be interesting to investigate whether these genes regulate a network of commensal-related coding sequences. Also, amongst the commensal gene set is the locus including the SlpA integrase that encodes the FxsA protein that affects phage T7 exclusion by the F plasmid [44], and a fimbrial subunit likely involved in adhesion (Pfam domain for Fimbrial proteins, representative CGSHi22121_609). Finally, there are at least three loci, each one with multiple adjacent proteins correlated with commensal strains, which contain two or more proteins annotated as phage-related proteins.

## Discussion

The SGH array provides an inexpensive high-throughput means to analyze the genomic content of any *H. influenzae* strain, by reporting on the possession of 2890 different gene clusters extracted from the WGS of 24 strains. Comparison of the WGS and SGH array data demonstrated the fidelity of the arrays. Comparisons of the hybridization values for the duplicated probe sets within a single array demonstrate their high reproducibility; and comparisons of the hybridization values among technical replicates (prepared with separate labeling, hybridizations, and scans) demonstrate high reproducibility across experiments.

We now have gene possession data on 210 *Haemophilus influenzae* strains, given the combination of the array data and WGS data. Analysis of the gene clusters from all strains reveals that only 23% (678/2890) of the supragenome is conserved across all strains. These data suggest that we are dealing with more than one species based on the Ahmed criteria [7] which states that if the addition of a single strain to what is an established and robust supragenome analysis (as we had for *H. influenzae*[2]) results in > 5% decrease in the core genome size then the last strain added is likely from a different species. If we exempt the HDHi and the other three core genome outliers, then 94.5% of strains contain 98% of all clusters. Based on our previous suggestion of using the core genome to define a species, we would argue that the differences observed for the HDHi are consistent with it forming a subspecies or even separate species. Twenty one of these novel strains were derived from longitudinal studies on two patients [43]. Analyses of these strains by Gilsdorf and colleagues for phenotypic characteristics routinely used to distinguish between *H. influenzae* and *H. haemolyticus* suggested that 19 out of 21 were not NTHi strains but instead met criteria for being classified as: 1) *H. haemolyticus;* 2) non-haemolytic *H. haemolyticus*; or 3) were chimeric strains with features of both *H. influenzae* and *H. haemolyticus*[46-48]. These strains were tested for the presence of *iga*, which encodes immunoglobulin A1 protease and for the presence of outer membrane protein P6 by reaction with 7F3 monoclonal antibody. They were also tested for hemolysis and porphyrin production. If they were positive for *iga* and P6 and negative for hemolysis and porphyrin, they were considered to be NTHi. Interestingly these two patients also carried strains in their pharynges that meet the criteria of *H. influenzae*, 14 of which were analyzed in this study using the SGH array. Three of the strains making up this novel lineage are from diverse sites, two were isolated in Australia from asymptomatic patients and one was isolated in Iowa from a patient with COPD. Two hypotheses can explain these results. First, this lineage is already widespread. Second, the bacteria in these patients were under similar fitness pressures to lose a particular set of genes. These hypotheses should be resolvable by whole genome sequence analysis which will reveal the sequence of alleles and any novel set of genes shared by these strains.

These data show the value of the SGH array for identifying core/distributed genes; grouping strains, and identifying new lineages/subspecies/species. It should be noted, however, that since the initial design did not include any representative strains from this novel lineage any lineage-specific genes will be missed by the array. Therefore, these results point us toward the sequencing of several representatives of this novel lineage and also determining their pathogenicity profile in our chinchilla model of otitis media and invasive disease [16,49,50], as a recent report has identified *H. haemolyticus* as being etiologically associated with invasive disease [51].

This analysis revealed that many genes are enriched in commensal strains (121 genes) while others are more commonly found in virulent strains (Additional file 2: Table S2). Most enriched genes have unknown function, and thus should provide a rich source for targeted studies to characterize novel functional categories associated with virulence and protection from virulence. In contrast, we did not observe a correlation between geographical location and gene content. For example the ten strains isolated in Pittsburgh (PittAA-PittJJ) are widely distributed throughout the phylogenetic tree. Similarly, the three strains isolated from Papua New Guinea, although they all fall into the same general branch, do not cluster closely together suggesting they are more similar to strains isolated in other locations than they are to each other.

The data collected as part of this study are relative to *Haemophilus sp*, but the same strategy could be used to design and produce supragenome arrays for any species for which the majority of the supragenome has been sampled by sequencing a reasonable sized subset of strains. Many human pathogens and commensals have extensive genome diversity, such as *Streptococcus pneumoniae, Escherichia coli, Garderella vaginallis,* and *Staphylococcus. aureus*[3,5,7,14]. This diversity is also observed in many environmental and plant pathogens such as *Pseudomonas aeruginosa, Xylella fastidiosus*, and *Bacillus cereus*[52,53]. The number of core genes as a percent of total genes varies extensively across species. *G. vaginalis*, *E.coli*, and *B. cereus* have high strain diversity, with only 27%, 35%, and 35% of total genes shared across all strains, respectively. In contrast, *M. tuberculosis* and *B. anthracis* have low strain diversity with ~91% of the supra (pan) genome shared across all strains [7]. Different species generate novel strain combinations by transformation, conjugation and/or transduction. *H. influenzae* are naturally competent, and a genome wide analysis of recombinants generated in the laboratory demonstrated that homologous recombination is the main mechanism generating strain diversity [54].

We hypothesize that the extensive differences in the size of the core relative to the supragenome reflect both the age and the evolutionary history of the taxonomic domain being studied as well as the mechanisms used for HGT. In this manner, species that cause chronic infections in highly variable niches, such as the dynamic microbiome present in the human nasopharynx, are likely to have been selected to increase strain diversification. Whereas, it is tempting to speculate that *B. anthracis,* which causes highly acute disease, and is not associated with long-term survival in the human host does not require a large distributed genome because of its 'hit and run' pathogenic profile, the larger truth is that it is not a species unto itself, but rather is a pathogenic clade of *B. cereus* which has a very large distributed genome [55]. Thus, *M. tuberculousis* is the sole species characterized to date that has a very limited supragenome which may relate to its very recent evolutionary origins, i.e. it simply has not had time to diversify as much as older species. This array provides a powerful strategy for accessing genome content of diverse strains within a species or related species, and thus provides a high value tool for research on human health and environmental science.

## Conclusions

The design and use of a custom SGH array based on a bacterial species supragenome modeled to contain the overwhelming majority (>85%) of non-rare genes provides a rapid and efficient means to screen large numbers of strains with regard to gene content [2,3,5]. These gene content data can then be parsed according to phenotype and subjected to statistical genetic tests to provide an unbiased method for identifying candidate genes for genotype-phenotype correlations. Thus, the method is independent of gene annotations and can point the way to useful new biology by identifying hypothetical genes of unknown function as being associated with given phenotypes. In the current analysis we employed this approach to identify candidate virulence genes for *H. influenzae.* Ongoing studies in our laboratories of two novel gene clusters that were targeted based, in part, on these technologies have been shown to indeed be virulence factors, and will be the subject of separate reports. Comprehensive SGH arrays also serve as useful tools for developing detailed visualizations and understandings of the genomic and phylogenetic relationships among strains, among groups of strains, and among closely related species. This aspect of the *Haemophilus* SGH described above revealed the existence of a novel clade within the species which we have termed the HDHi.

## Ethics statement

We used only purified clonal bacterial isolates, and did not need to perform isolations from patient derived samples. Therefore no specific ethics approval or consent was necessary.

## Competing interests

GDE and FZH are authors on a US patent application (No. 61/233,642) “Use of Distributed Gene Data for Development of Strain-specific Bacterial Diagnostics”.

## Authors’ contributions

RAE isolated DNA and carried out the array experiments, conceived the project and wrote the manuscript. NLH designed the SGH Array, conceived the project and wrote the manuscript. JPE created graphical representations of the data. BAJ isolated DNA and carried out the array experiments; conceived the project and wrote the manuscript. MED designed the SGH Array. AA isolated DNA and carried out the array experiments. EP isolated DNA and carried out the array experiments. MPS created graphical representations of the data. JRG collected and contributed bacterial isolates. LZ collected and contributed bacterial isolates. AS collected and contributed bacterial isolates. TFM collected and contributed bacterial isolates. SS collected and contributed bacterial isolates. KS collected and contributed bacterial isolates. JCP conceived the project. FZH conceived the project. GDE conceived the project and wrote the manuscript. All authors read and approved the final manuscript.

## Supplementary Material

Additional file 1: Table S1 List of all strains used in this study. Click here for file

Additional file 2: Table S2 List of 149 gene clusters enriched in either the 118* virulent strains or the 65 commensal strains. Click here for file

Additional file 3: Table S3 List of methylases enriched in either virulent or commensal strains. Click here for file
